# The remodeling of metabolic brain pattern in patients with extracranial diffuse large B-cell lymphoma

**DOI:** 10.1186/s13550-023-01046-6

**Published:** 2023-10-30

**Authors:** Junyi Liu, Ming Tang, Dongling Zhu, Ge Ruan, Sijuan Zou, Zhaoting Cheng, Xiaohua Zhu, Yuankai Zhu

**Affiliations:** 1 Department of Nuclear Medicine and PET Center, Tongji Hospital, Tongji Medical College, Huazhong University of Science and Technology, No. 1095 Jiefang Ave, Wuhan, 430030 China; 2 Department of Radiology, Hospital, Hubei University, Wuhan, 430062 China

**Keywords:** Diffuse large B-cell lymphoma (DLBCL), Positron emission tomography, Metabolic brain pattern, Tumor burden, Staging, Prognosis

## Abstract

**Background:**

Owing to the advances in diagnosis and therapy, survival or remission rates for lymphoma have improved prominently. Apart from the lymphoma- and chemotherapy-related somatic symptom burden, increasing attention has been drawn to the health-related quality of life. The application of ^18^F-fluorodeoxyglucose positron emission tomography-computed tomography (^18^F-FDG PET/CT) has been routinely recommended for the staging and response assessment of FDG-avid lymphoma. However, up till now, only a few researches have investigated the brain metabolic impairments in patients with pre-treatment lymphoma. The determination of the lymphoma-related metabolic brain pattern would facilitate exploring the tailored therapeutic regimen to alleviate not only the physiological, but also the psychological symptoms. In this retrospective study, we aimed to establish the diffuse large B-cell lymphoma-related pattern (DLBCLRP) of metabolic brain network and investigate the correlations between DLBCLRP and several indexes of the staging and response assessment.

**Results:**

The established DLBCLRP was characterized by the increased metabolic activity in bilateral cerebellum, brainstem, thalamus, striatum, hippocampus, amygdala, parahippocampal gyrus and right middle temporal gyrus and by the decreased metabolic activity in bilateral occipital lobe, parietal lobe, anterior cingulate gyrus, midcingulate cortex and medial frontal gyrus. Significant difference in the baseline expression of DLBCLRP was found among complete metabolic response (CMR), partial metabolic response (PMR) and progressive metabolic disease (PMD) groups (*P* < 0.01). DLBCLRP expressions were also significantly or tended to be positively correlated with international prognostic index (IPI) (*r*_*s*_ = 0.306, *P* < 0.05), lg(total metabolic tumor volume, TMTV) (*r* = 0.298, *P* < 0.05) and lg(total lesion glycolysis, TLG) (*r* = 0.233, *P* = 0.064). Though no significant correlation of DLBCLRP expression was found with Ann Arbor staging or tumor SUV_max_ (*P* > 0.05), the post-treatment declines of DLBCLRP expression were significantly positively correlated with Ann Arbor staging (*r*_*s*_ = 0.284, *P* < 0.05) and IPI (*r*_*s*_ = 0.297, *P* < 0.05).

**Conclusions:**

The proposed DLBCLRP would lay the foundation for further investigating the cerebral dysfunction related to DLBCL itself and/or treatments. Besides, the expression of DLBCLRP was associated with the tumor burden of lymphoma, implying a potential biomarker for prognosis.

## Background

Lymphoma refers to a type of cancer derived from lymphatic system. Based on the types of involved lymphocyte cells, lymphoma can be roughly divided into two main types: Hodgkin’s lymphoma (HL) and non-Hodgkin’s lymphoma (NHL) [1]. NHL accounted for approximately 2.82% of 19.3 million new cancer cases and 2.63% of 9.9 million cancer deaths worldwide in 2020 [2]. As a phenotypically and genetically heterogeneous disorder, diffuse large B-cell lymphoma (DLBCL) was one of the most common subtypes of NHL [3].

Owing to the advances in diagnosis and therapy, survival or remission rates for lymphoma have improved prominently. Apart from the lymphoma- and chemotherapy-related somatic symptom burden, increasing attention has been drawn to the health-related quality of life [4, 5]. In particular, the presence of cognitive impairment, negative psychological status (e.g., anxiety, depression and committing suicide) or other emotional symptoms among patients with hematological cancer was supposed to be more likely than those with solid tumor [6–9]. Furthermore, psychological complications were common among NHL patients, especially for the more aggressive subtypes [10]. Though these brain dysfunctions were assumed to be mainly associated with chemotherapy neurotoxicity, the disease status of non-central nervous system (non-CNS) cancer itself was also considered to be relevant [11, 12]. Knowledge about the underlying mechanisms for brain dysfunction induced by non-CNS lymphoma itself, would further facilitate the better assessment of exclusive detrimental effect related to chemotherapy or other treatments and, even more importantly, allow for developing tailored therapeutic regimen to alleviate not only the physiological, but also the psychological symptoms.

The application of ^18^F-fluorodeoxyglucose positron emission tomography-computed tomography (^18^F-FDG PET/CT) has been routinely recommended for the staging and response assessment of FDG-avid lymphoma, especially DLBCL [13, 14]. Besides, ^18^F-FDG PET/CT imaging could also be applied for the noninvasive appraisal of altered brain functions from the perspective of glucose metabolism. An increasing number of ^18^F-FDG PET studies revealed that chemotherapy would lead to regional brain glucose metabolic abnormalities among lymphoma patients [15–18]. However, up till now, only a few researches have investigated the brain metabolic impairments in patients with pre-treatment lymphoma [19–21].

Compared with the normal control, lymphoma patients prior to the initiation of chemotherapy showed regional hypometabolism primarily in the parieto-occipital lobes [20]. However, it is still up for debate whether or not these cerebral metabolic abnormalities related to the disease status of lymphoma will change after receiving chemotherapy [19, 21]. Moreover, higher FDG uptake in basal ganglia and thalamus or lower FDG uptake in cerebellum relative to liver based on pre-treatment ^18^F-FDG PET data was supposed to have the potential for predicting a worse prognosis in patients with DLBCL or extranodal natural killer/T-cell lymphoma [22, 23]. All of these brain metabolic parameters were derived from univariate methods accounting for only the intensity or magnitude. To the best of our knowledge, lymphoma-related pattern of metabolic brain network has not been validated.

The primary aim of this study was to establish and validate the DLBCL-related pattern (DLBCLRP) of metabolic brain network. In order to analyze discrepancies in the expressions of lymphoma-related pattern between DLBCL and HL, the expressions of DLBCLRP in HL patients were also investigated. Moreover, the correlations between DLBCLRP and several indexes of the staging and response assessment were further explored.

## Materials and methods

### Subjects

Lymphoma patients referred for whole-body ^18^F-FDG PET/CT imaging from July 2014 to June 2021 were retrospectively investigated. Clinical information was extracted through electronic medical record system. Inclusion criteria for DLBCL group were as follows: (1) patients with histologically confirmed DLBCL without CNS involvement; (2) available baseline whole-body ^18^F-FDG PET/CT scan before initiating R‐CHOP or R‐CHOP‐like (R-DA-EPOCH, R-CDOP or R-GCVP) therapy (R = Rituximab; C = Cyclophosphamide; H = Hydrochloride, O = Oncovin; P = Prednisone; DA = Dose adjusted; E = Etoposide; D = Doxorubicin; G = Gemcitabine and V = Vincristine); (3) receiving a post-treatment ^18^F-FDG PET/CT scan at interim or end of chemotherapy; (4) available detailed clinical data related to DLBCL; (5) lymphoma lesions with hypermetabolism and (6) age > 18 years. Patients were excluded if they had any brain structural abnormality or any past history of neuropsychiatric disorder. Other exclusion criteria consisted of the intracranial lymphoma infiltration and the incomplete cerebrum covered in the field of view. Since patients were excluded if they had any brain structural abnormality or intracranial lymphoma infiltration, neither high-dose methotrexate (HD-MTX) nor intrathecal (IT) MTX therapy was applied to any patients introduced in this study. Patients with MTX preventive therapy were also excluded, in order to ensure consistency in treatment regimens as far as possible. Besides, no patient received autologous or allogeneic stem cell transplant treatment before the interim ^18^F-FDG PET/CT scan. Finally, 64 patients (28 females; median [interquartile range, IQR] age, 51 [37–61] years) were introduced to form the DLBCL group. Furthermore, the control group including 49 adult subjects (27 females; median [IQR] age, 49 [43–54] years) dedicated for the establishment of DLBCLRP, was screened from patients without any brain pathology as described in our previous works [24, 25].

In order to further verify the established DLBCLRP, another 39 DLBCL patients (24 females; median [IQR] age, 51 [42–64] years) from the same institution were also included as the validation group (Table 1). Besides, the expressions of DLBCLRP were tested in 31 HL patients (15 females; median [IQR] age, 31 [22–52] years) as well. The inclusion and exclusion criteria for the validation or HL group were the same as described above, except the lack of post-treatment PET/CT data or/and the diagnosed HL. This study design was approved by the Institutional Review Board, and the exemption from informed consent was obtained as well.

**Table 1:** The demographics and clinical characteristics of DLBCL and validation groups

Clinical characteristics	DLBCL (*n* = 64)	Validation (*n* = 39)	*P* value
Gender			
Female/male	28/36	24/15	0.105*
Age (years)	51 (37–61)	51 (42–64)	0.688
Ann Arbor staging			0.258
I	2	4	
II	28	10	
III	19	10	
IV	15	15	
IPI			0.327
0	10	5	
1	19	6	
2	14	15	
3	15	8	
4	4	4	
5	2	1	
Lymphoma SUV_max_	23.69 ± 6.94	22.14 ± 6.62	0.264
Lg(TMTV)	1.98 ± 0.57	1.94 ± 0.69	0.771
Lg(TLG)	3.10 ± 0.59	3.03 ± 0.70	0.570

DLBCL = diffuse large B-cell lymphoma; IPI = international prognostic index; TMTV = metabolic tumor volume and TLG = total lesion glycolysis; * χ^2^ = 3.068

### PET images acquisition

Routine whole-body ^18^F-FDG PET/CT scans were conducted through a clinical PET/CT scanner (Discovery 690 Elite, GE Medical Systems, USA), at 60 min after intravenous injection of radiotracer (3.7 MBq/kg). All PET data were acquired in three-dimensional (3D) mode with a separated brain scan. The voxel size of reconstructed whole-body or brain PET images was 3.65 × 3.65 × 3.27 mm^3^ or 1.56 × 1.56 × 3.27 mm^3^, respectively, based on a fully 3D iterative reconstruction algorithm of SharpIR + VUE point HD.

### PET images analyses

#### Establishing and verifying the DLBCLRP

All the reconstructed brain PET data were spatially normalized into the default brain template provided by the statistical parametric mapping 8 (SPM8) software package (Welcome Trust Centre for Neuroimaging, London, UK; http://www.fil.ion.ucl.ac.uk/spm). The voxel size of these normalized images was set at 2 × 2 × 2 mm^3^ and followed by smoothing process with an 10-mm isotropic Gaussian kernel [26]. Considering the intrinsically non-quantitative nature of these PET images, voxel-wise intensity normalization was conducted by setting the whole brain as the reference.

The metabolic brain network pattern for DLBCLRP was determined by using the Scan Analysis and Visualization Processor (ScAnVP) software package (Center for Neuroscience, Feinstein Institute for Medical Research, NY; http://www.feinsteinneuroscience.org). The embedded algorithm with scaled subprofile model of principal component analysis (SSM-PCA) was applied, to achieve a maximum distinction in the expression of DLBCLRP between the control and DLBCL groups [24].

Based on the established metabolic brain network pattern, the Z-transformed score of principal components expression for each DLBCL patient was obtained, to facilitate the subsequent statistical analyses. Moreover, the expressions of DLBCLRP in subjects from the validation and HL groups were also calculated by assessing their topographic profiles and then transformed into Z score.

#### Metabolic features of lymphoma

The maximum standardized uptake value (SUV_max_) of lymphoma lesions for each patient was recorded through whole-body PET/CT images. Besides, baseline staging with Ann Arbor system (Stages I–IV) was performed based on the distribution of lymphoma infiltration [27, 28]. International prognostic index (IPI) served as the baseline prognostic assessment (score from 0 to 5), considering the features of age, Ann Arbor staging, extranodal involvement, serum lactate dehydrogenase level and performance status assessed by Eastern Cooperative Oncology Group (ECOG) scale [29]. This ECOG performance status scale has been widely used to assess the functional status of cancer patient [30].

The Deauville 5-point scale (5-PS) was used for the response assessment of each DLBCL patient [14]. Based on the assessed score and the alteration in the degree of FDG uptake from baseline at post-treatment, the PET/CT-based response appraisal was further divided into four categories: complete metabolic response (CMR), partial metabolic response (PMR), no metabolic response (NMR) and progressive metabolic disease (PMD). These response assessments of DLBCL were classified based on the new Lugano classification criterion [14, 27].

The whole-body ^18^F-FDG PET data were transferred into the LIFEx platform (version 6.30; https://www.lifexsoft.org/). Using the threshold at the 41% of focal lesion SUV_max_, 3D isocontour volume of interest (VOI) was semi-automatically delineated for each FDG-avid lesion. However, the tissues of brain, myocardium, bladder and kidney with relatively high physiological FDG uptake were manually excluded from the VOI definition, except for the focal uptake in bone marrow, spleen or liver. Then, quantitative measures of tumor burden were assessed with the parameters of metabolic tumor volume (MTV) and total lesion glycolysis (TLG) [31]. The semi-automatically collected total MTV (TMTV) for each patient represents the sum of segmented VOIs, while the TLG was calculated as the product of the TMTV and the value of SUV_mean_. The metabolic parameters of brain SUV_max_, SUV_mean_ and total brain glycolysis (TBG) were also analyzed to evaluate the brain FDG uptake level. Similar to the formula for TLG, the value of TBG was calculated as the product of the total brain metabolic volume and the value of brain SUV_mean_ [19].

### Statistical analysis

Statistical analyses were conducted by using SPSS software (version 25.0; IBM SPSS Statistics) and GraphPad Prism (Version 5.01 for Windows, GraphPad Software, San Diego, California, USA, www.graphpad.com). Group differences between two groups for continuous variables were analyzed by the Mann–Whitney test or two-sample *t*-test (normal distribution with equal variances). Besides, the paired *t*-test was conducted to assess the differences in pattern expression between baseline and post-treatment. Group differences among three groups were analyzed by the Kruskal–Wallis test followed by post hoc Dunn’s multiple comparisons test or the one-way ANOVA followed by post hoc Bonferroni multiple comparisons test (normal distribution with equal variances). Categorical variable of gender was analyzed using the Pearson chi-square test. Correlation analysis was conducted by Pearson or Spearman correlation coefficients as appropriate. The differences in correlation coefficients were compared using MedCalc statistical software (Version 11.4.2.0). *P* value < 0.05 was considered statistically significant.

## Results

### The demographics and clinical characteristics

There was no significant difference in age (*P* = 0.688), gender (χ^2^ = 3.068, *P* = 0.105), Ann Arbor staging (*P* = 0.258), IPI (*P* = 0.327), lymphoma SUV_max_ (*P* = 0.264), lg(TMTV) (*P* = 0.771) or lg(TLG) (*P* = 0.570) between DLBCL and validation groups (Table 1). In addition, no significant difference in age (*P* = 0.131) or gender (χ^2^ = 1.432, *P* = 0.259) was found between control and DLBCL groups. Though no significant difference in gender (χ^2^ = 0.181, *P* = 0.826) was found between DLBCL and HL groups, HL group was significantly younger than DLBCL group (*P* < 0.001).

### Metabolic brain network analysis for DLBCL

**Fig. 1:**
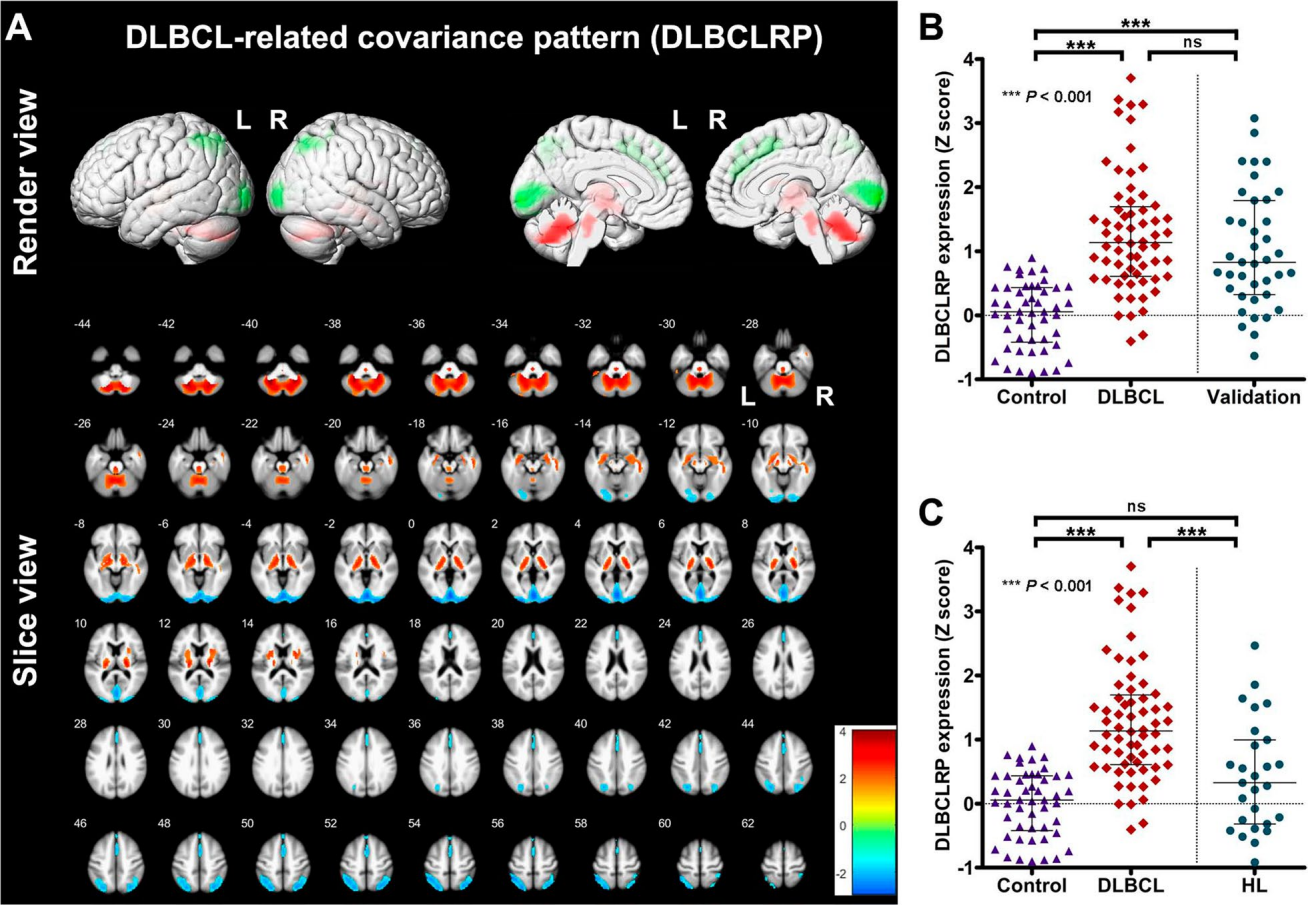
Metabolic brain network analysis for DLBCL. **A** DLBCL-related covariance pattern (DLBCLRP) was established and displayed in the render and slice views. Voxels with increased metabolic activity were shown in the red color, while those with decreased metabolic activity in the green or blue color. **B** Pattern expressions of DLBCLRP were compared among control, DLBCL and validation groups. **C** DLBCLRP expressions in HL group were compared with those in control and DLBCL groups

The established DLBCLRP was characterized by the increased metabolic activity in bilateral cerebellum, brainstem, thalamus, striatum, hippocampus, amygdala, parahippocampal gyrus and right middle temporal gyrus (Fig. 1A). The associated brain regions with decreased metabolic activity were located at bilateral occipital lobe, parietal lobe, anterior cingulate gyrus, midcingulate cortex and medial frontal gyrus. DLBCLRP expressions in DLBCL group were significantly positively correlated with their age (*r* = 0.259, *P* = 0.039, Spearman correlation coefficients). Besides, male patients (median [IQR], 1.323 [0.779–2.171]) had significantly higher DLBCLRP expressions than female patients (median [IQR], 0.880 [0.532–1.493]; *P* = 0.038).

Significant differences in the expression of DLBCLRP were found among control, DLBCL and validation (or HL) subjects (*P* < 0.001), and the *post hoc* Dunn’s tests are shown in Fig. 1B or Fig. 1C. Both DLBCL (1.28 ± 0.93) and validation (1.01 ± 0.91) groups had significantly higher DLBCLRP expressions than control group (6.12 × 10^–5^ ± 0.51; *P* < 0.001). However, no significant difference in the pattern expression was found between DLBCL and validation groups or between control and HL (0.42 ± 0.82) groups (*P* > 0.05). Compared with DLBCL group, HL group showed significantly lower expression of DLBCLRP (*P* < 0.001).

**Fig. 2:**
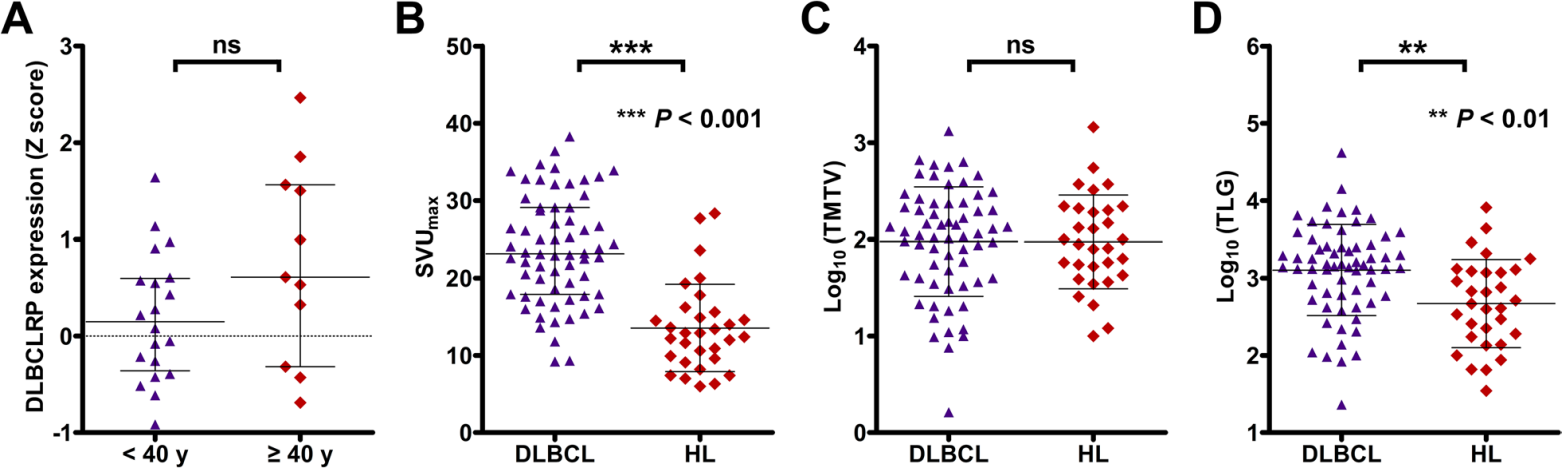
The pattern expressions of DLBCLRP in HL patients. **A** Comparison of DLBCLRP expressions between HL patients < 40 y and those ≥ 40 y. **B** Comparison of tumor SUV_max_ between DLBCL and HL patients. **C** Comparison of TMTV in the form of lg transformation between DLBCL and HL patients. **D** Comparison of TLG in the form of lg transformation between DLBCL and HL patients

As shown in Fig. 2A, no significant difference in the pattern expression was found between HL patients < 40 yrs and those ≥ 40 yrs (0.20 ± 0.66 vs. 0.77 ± 1.02; *P* > 0.05). The values of lymphoma SUV_max_ in HL patients were significantly lower than those in DLBCL patients (13.55 ± 5.66 vs. 23.69 ± 6.94; *P* < 0.001; Fig. 2B). Though no significant difference in the value of lg(TMTV) was found between DLBCL and HL groups (1.98 ± 0.57 vs. 1.97 ± 0.48; *P* > 0.05; Fig. 2C), HL patients had significantly lower lg(TLG) than DLBCL patients (2.67 ± 0.57 vs. 3.10 ± 0.59; *P* < 0.01; Fig. 2D).

### Correlation between baseline DLBCLRP expressions and indicators for lymphoma assessment

**Fig. 3:**
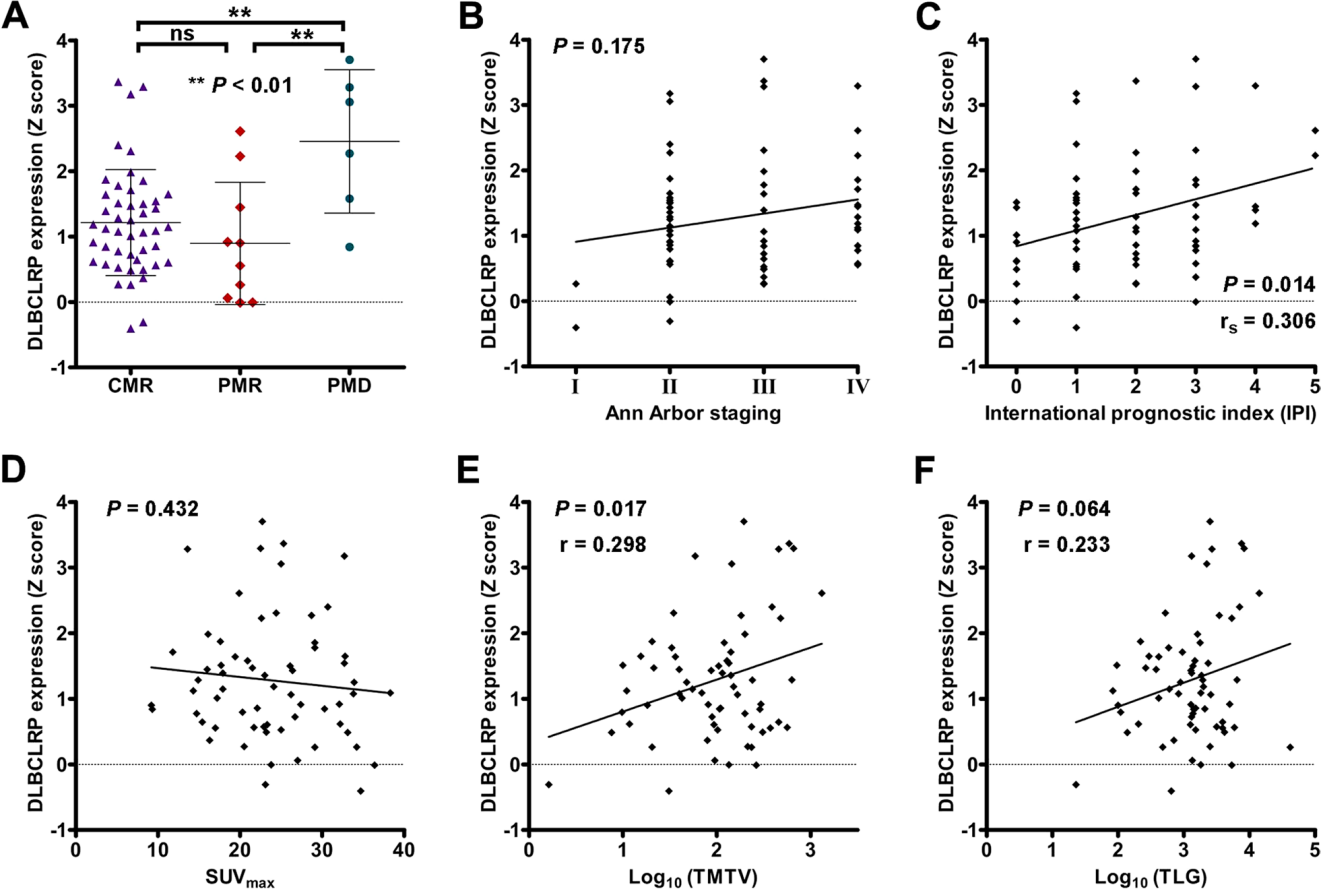
Correlation between baseline DLBCLRP expressions and indicators for lymphoma assessment. **A** Pattern expressions of DLBCLRP were compared among CMR, PMR and PMD groups. Correlations of DLBCLRP expressions with Ann Arbor staging, IPI, tumor SUV_max_, lg(TMTV) and lg(TLG) were analyzed using Spearman (**B** and **C**) or Pearson (**D**-**F**) correlation coefficients, respectively

Significant differences in the expression of DLBCLRP were found among CMR, PMR and PMD groups (*P* < 0.01; Fig. 3A). Bonferroni multiple comparisons indicated that PMD group (2.46 ± 1.10) had significantly higher pattern expressions than both CMR (1.21 ± 0.81) and PMR (0.90 ± 0.94) groups (*P* < 0.01). However, no significant difference in the pattern expression was found between CMR and PMR groups (*P* > 0.05), and no significant correlation of DLBCLRP expression was found with Ann Arbor staging or tumor SUV_max_ (Fig. 3B and D; *P* > 0.05). Besides, DLBCLRP expressions were significantly positively correlated with IPI (*r*_*s*_ = 0.306, *P* < 0.05; Fig. 3C) and lg(TMTV) (*r* = 0.298, *P* < 0.05; Fig. 3E), and tended to be positively correlated with lg(TLG) (*r* = 0.233, *P* = 0.064; Fig. 3F).

### The alteration of DLBCLRP expressions after treatment

**Fig. 4:**
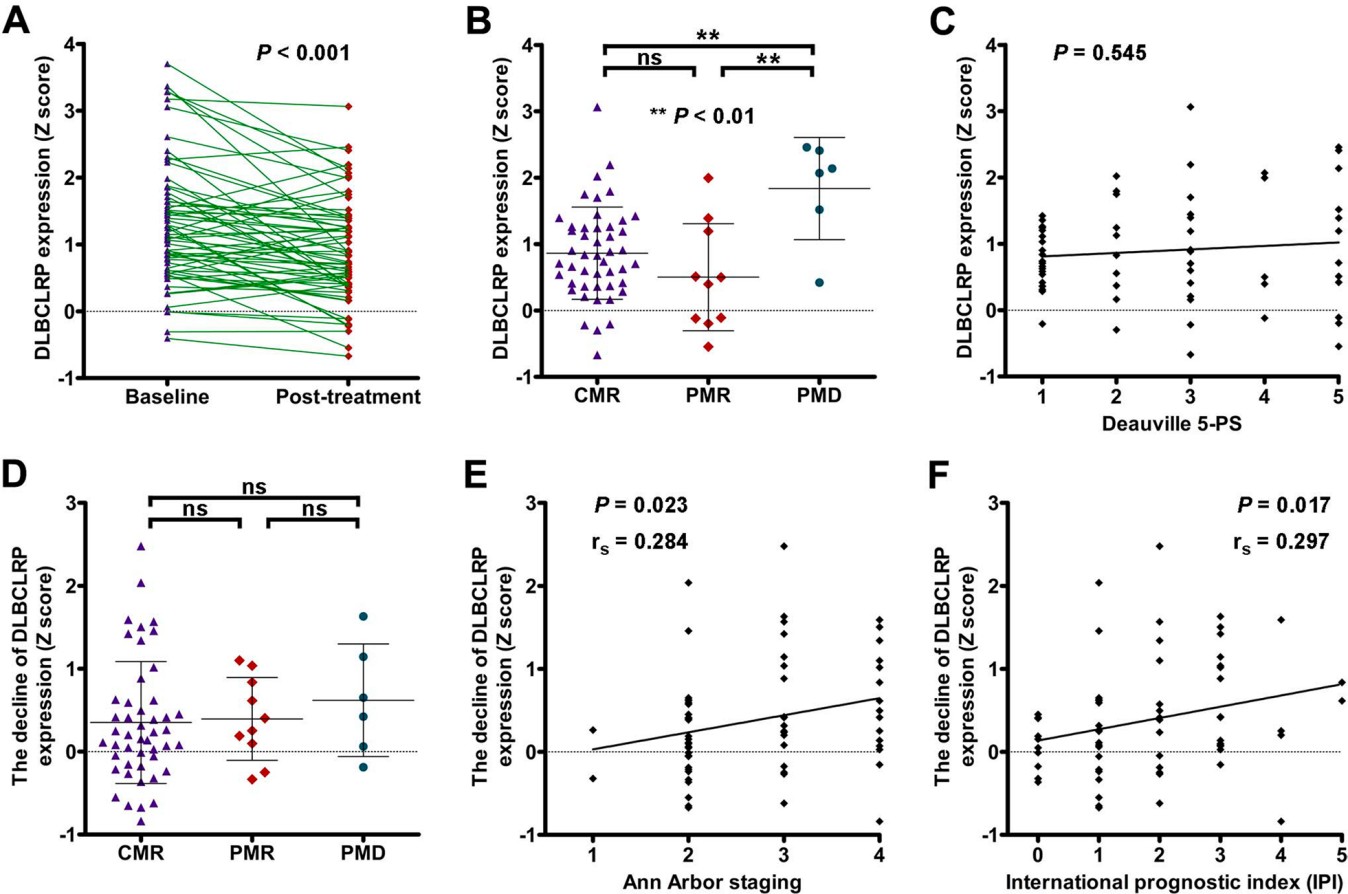
The alteration of DLBCLRP expressions after treatment and its correlation with indicators for lymphoma assessment. **A** DLBCLRP expressions of post-treatment were paired compared with those of baseline. **B** Post-treatment DLBCLRP expressions were compared among CMR, PMR and PMD groups. **C** Correlation of post-treatment DLBCLRP expressions with Deauville 5-point scale (5-PS) was analyzed using Spearman correlation coefficients. **D** The post-treatment declines of DLBCLRP expression were compared among CMR, PMR and PMD groups. Besides, the correlations of post-treatment declines of DLBCLRP expression with Ann Arbor staging (**E**) and IPI (**F**) were analyzed using Spearman correlation coefficients

There was a significant reduction in the DLBCLRP expressions of post-treatment, compared with those of baseline (0.90 ± 0.78 vs. 1.28 ± 0.93; *P* < 0.001; Fig. 4A). Significant differences in the post-treatment expression of DLBCLRP were found among CMR, PMR and PMD groups (*P* < 0.01; Fig. 4B). Bonferroni multiple comparisons indicated that PMD group (1.84 ± 0.77) had significantly higher post-treatment pattern expressions than both CMR (0.86 ± 0.70) and PMR (0.50 ± 0.81) groups (*P* < 0.01). There was no statistically significant correlation between post-treatment DLBCLRP expressions and Deauville 5-PS (Fig. 4C; *P* > 0.05). No significant difference in the post-treatment declines of DLBCLRP expression was found among CMR, PMR and PMD groups (Fig. 4D; *P* > 0.05). However, the post-treatment declines of DLBCLRP expression were significantly positively correlated with Ann Arbor staging (*r*_*s*_ = 0.284, *P* < 0.05; Fig. 3E) and IPI (*r*_*s*_ = 0.297, *P* < 0.05; Fig. 3F). In the DLBCL group, 59 patients were treated with R‐CHOP, while other five patients with R‐CHOP‐like (three patients with R-DA-EPOCH, one patient with R-CDOP and one patient with R-GCVP) therapy. No significant difference in the post-treatment decline of DLBCLRP expression was found between patients receiving R‐CHOP therapy and those receiving R‐CHOP‐like therapy (0.40 ± 0.71 vs. 0.18 ± 0.40; *P* = 0.662; Mann–Whitney test).

### The FDG uptake level of whole brain

As shown in Table 2, the brain SUV_max_ (*r* = − 0.560), brain SUV_mean_ (*r* = − 0.612) and TBG (*r* = − 0.595) were significantly negatively correlated with the DLBCLRP expression in DLBCL patients (*P* < 0.001). Besides, the brain SUV_max_, brain SUV_mean_ and TBG were also significantly negatively correlated with both the lg(TMTV) (*r* = − 0.345, − 0.375 and − 0.402, respectively; *P* < 0.01) and lg(TLG) (*r* = − 0.266, − 0.279 and − 0.302, respectively; *P* < 0.01). However, there was no statistically significant correlation between these parameters of brain FDG uptake and lymphoma SUV (maximum or mean), except that the TBG was significantly positively correlated with lymphoma SUV_mean_ (*r* = 0.257; *P* < 0.05). Since the TLG was calculated as the product of the TMTV and the value of lymphoma SUV_mean_, the correlation coefficient between TBG and lg(TMTV) was further compared with that between TBG and lg(TLG). By using MedCalc statistical software, no significant difference was found between the two correlation coefficients (Z = − 0.613, *P* = 0.528).

**Table 2:** Correlation between the parameters of brain FDG uptake and indicators for lymphoma assessment

Parameters	Pearson correlation coefficients				Spearman correlation coefficients			
	Z score	Lg(TMTV)	Lg(TLG)	^#^SUV_max_	^#^SUV_mean_	^†^Staging	IPI	5-PS
Brain SUV_max_	− 0.560***	− 0.345**	− 0.266*	ns	ns	− 0.312*	− 0.442***	ns
Brain SUV_mean_	− 0.612***	− 0.375**	− 0.279*	ns	ns	− 0.305*	− 0.419**	ns
TBG	− 0.595***	− 0.402**	− 0.302*	ns	0.257*	− 0.293*	− 0.426**	− 0.267*

5-PS = Deauville 5-PS; IPI = international prognostic index; TBG = total brain glycolysis; TLG = total lesion glycolysis; TMTV = total metabolic tumor volume and ns = no significant; ^†^ Ann Arbor staging; ^#^ SUV of tumor; *P < 0.05; **P < 0.01 and ***P < 0.001

In regard to the indicators for lymphoma assessment, the brain SUV_max_, brain SUV_mean_ and TBG were significantly negatively correlated with both the Ann Arbor staging (*r* = − 0.312, − 0.305 and − 0.293, respectively; *P* < 0.05) and IPI (*r* = − 0.442, − 0.419 and − 0.426, respectively; *P* < 0.01). Though no statistically significant correlation was found between SUV (maximum or mean) and Deauville 5-PS (*P* > 0.05), the TBG was significantly negatively correlated with Deauville 5-PS (*r* = − 0.267; *P* < 0.05).

## Discussion

The current study provided a fresh perspective for the altered metabolic brain network in DLBCL. The established pattern of brain network related to DLBCL could be verified through validation group and was completely different from that of HL. This DLBCLRP was characterized by the increased metabolic activity in brain regions rarely involving cerebrum. The expression of DLBCLRP was associated with the tumor burden of lymphoma, implying a potential biomarker for staging and prognosis. These findings about brain dysfunction induced by non-CNS lymphoma itself, would also facilitate the further exploration of exclusive detrimental effect related to chemotherapy or other treatments.

It was suggested that not only chemotherapy, but also cancer itself could disrupt the functional connectivity measured with resting- or task-state functional magnetic resonance imaging (fMRI) [11, 32, 33]. For the patients with breast cancer before receiving any treatment, the lower whole-brain functional connectivity, the greater decline in cognitive function [33]. These aberrant functional connectivities were also considered to be relevant to the memory impairment and fatigue [11, 32]. The presence of neuropsychiatric symptoms among patients with hematological cancer was supposed to be more likely than those with solid tumor [6–9]. Thus, it is necessary and critical to investigate the underlying mechanisms for brain dysfunction induced by non-CNS lymphoma itself. However, evidence for the association between brain function and metabolic network in patients with extracranial DLBCL is still lacking. Only a few studies have explored the lymphoma-related metabolic features, by using univariate methods accounting for merely the intensity or magnitude of ^18^F-FDG PET data [20, 34–36]. The glucose metabolic levels of bilateral frontal, temporal and parietal lobes were negatively correlated with the lymphoma burden [20, 34]. But the discrepancy in metabolic features between different types of lymphoma was not further discussed. Hypometabolism might be localized prominently at bilateral occipital lobes for the young HL patients [35]. DLBCL with immune effector cell-associated neurotoxicity syndrome after receiving chimeric antigen receptor T-cell therapy showed a diffuse brain hypometabolism [36]. Moreover, in T-cell lymphoma patient, global hypometabolism and its associated neuropsychiatric symptoms can be reversed by administrating glucose [37]. To better understand the alteration of metabolic brain network in DLBCL, multivariate decomposition technique with SSM-PCA was introduced to establish DLBCLRP in this study.

The verified pre-treatment DLBCLRP was characterized by the relatively increased metabolic activity in bilateral cerebellum, brainstem, thalamus, striatum, hippocampus, amygdala, parahippocampal gyrus and right middle temporal gyrus. Put another way, hardly any cerebrum was involved in this compensatory increase in metabolic network. Based on the parameters of brain SUV_max_, SUV_mean_ and TBG, however, the FDG uptake level of whole brain in DLBCLRP patients was reduced compared with that of the control [34]. This metabolic decline might be attributed to the competition from the tumor tissue with huge energy demand [21]. We speculated that the brain basic functions (e.g., heartbeat and breathing) would be reserved as priority to cope with this relative energy shortage. Accordingly, DLBCLRP demonstrates compensatory and relatively elevated glucose consumption almost exclusively located at lower-level brain structures, including cerebellum, brainstem and diencephalon.

Accounting for two-thirds of the total brain volume, the cerebrum should be responsible for processing a series of complex brain functions, such as emotion, language, cognition and so on [38–40]. Principal component analysis in the current study also revealed that metabolic activity remained relative stable in the frontal and temporal lobes, but declined in the parietal and occipital lobes. Thus, the latter brain regions involving relatively less significant functions were considered more likely to be sacrificed in the remodeling of metabolic brain pattern for DLBCLRP. The occipital lobe was considered as the center for visual processing, while the parietal lobe, the center for sensation information processing related to cognition and speech. Besides, the anterior cingulate gyrus, midcingulate cortex and the adjacent medial frontal cortex should be responsible for regulating the emotion, memory storage and behavior. Consequently, these dysfunctional brain regions might be involved in the formation of DLBCLRP-associated neuropsychiatric symptoms, including visual hallucinations, depression, anxiety, cognitive and speech dysfunctions, fatigue, etc. [4, 10, 37, 41].

To determine whether there is a difference in the patterns of metabolic brain network between DLBCL and HL, the expressions of DLBCLRP in HL patients were also investigated. We found that the established DLBCLRP could not be applied to the patients with HL. Though HL group was significantly younger than DLBCL group in our study, no significant difference in the pattern expression was found between HL patients < 40 yrs and those ≥ 40 yrs. Therefore, the discrepancy in the patterns of metabolic brain network between two types of lymphoma should not be attributed to the disparity in their age of onset [2]. Though no significant difference in the TMTV was found between DLBCL and HL groups, HL patients had significantly lower SUV_max_ and TLG than DLBCL patients. Thus, it was speculated that the expression of DLBCLRP might be associated with the tumor burden of lymphoma.

To confirm this assumption, correlation between the expression of DLBCLRP and the parameters of tumor burden (TMTV and TLG) was further explored. The Z scores of pattern expression were significantly positively correlated with TMTV and tended to be positively correlated with TLG in the form of log transformation. Moreover, our results also suggested that the higher level of whole-brain FDG uptake was associated with the lower pattern expression, TMTV and TLG. In consideration of the competition for capturing glucose between the tumor and the brain tissue, the tumor burden of lymphoma might indirectly affect the expression of DLBCLRP via whittling down the whole-brain “input” of glucose/FDG. Then, the metabolic brain network would undergo remodeling to accommodate the reduced energy supply as discussed above. Moreover, there was an inverse relationship between plasma glucose levels and brain FDG uptake [42]. Based on this assumption, the declined brain “input” of glucose and its associated DLBCLRP might be reversed by the administration of glucose supplementation [37].

Considering no significant difference in age and gender between DLBCL and control groups, this DLBCLRP can still be established, but might vary in some extent with age or gender. One possible explanation might be the brain compensatory function declining with age; subsequently, the metabolic features and network of DLBCLRP are prone to be tempted among the elderly [43, 44]. Both brain function and its glucose metabolism could be altered in a sex-dependent manner [45]. This feature may be attributed to the sex differences in genes, transcriptomes and hormones [46]. More detailed investigations are needed to verify the necessity to establish age-specific or gender-specific DLBCLRP. Furthermore, we are very curious to further explore the correlation and disparity between DLBCLRP and senile neurodegenerative diseases-related brain metabolic pattern.

By using the principal component analysis approach, the disease-related metabolic covariance patterns have been explored in a number of neurodegenerative diseases, including Parkinson's disease (PD), Alzheimer's disease (AD), dementia with Lewy bodies (DLB), mild cognitive impairment (MCI), fronto-temporal dementia (FTD), REM sleep behavior disorder (RBD), spinocerebellar ataxia (SCA), amyotrophic lateral sclerosis (ALS) and Parkinson plus syndromes (PP) [47–55]. Among these verified disease-related metabolic brain pattern, the most well-known PD-related pattern (PDRP) was characterized by relative hypermetabolism in pallidothalamic and pontocerebellar regions, accompanied by relative hypometabolism in the premotor and parieto-occipital association regions [56]. This spatial covariance PDRP exhibited a high degree of repeatability in PD population [26, 47]. Beyond the differential diagnosis of PD, the expression of PDRP was found to be correlated to motor manifestations and could serve as a biomarker for assessing and monitoring the therapeutic efficacy in PD patients [57]. However, in addition to the DLBCL-specific changes, a wide range of overlapped brain regions was detected between the canonical PDRP and our proposed DLBCLRP. Other neurodegenerative disorders would exhibit greater variability with DLBCL in the disease-related metabolic pattern [49, 51–53]. Furthermore, though we found that the established DLBCLRP could not be applied in another common type of lymphoma, HL, it is still unable to easily draw such a conclusion that this metabolic brain pattern can serve as a robust metabolic marker for the diagnosis of DLBCL. Further straightforwardly investigating the similarities and differences in metabolic brain pattern, and in pattern-associated determinants between DLBCL and PD (or other neurodegenerative disorders), would be very attractive.

Since the expression of DLBCLRP could be associated with the tumor burden of lymphoma, another implication for the establishment of DLBCLRP was whether it could serve as a potential metabolic brain biomarker for the prognosis of DLBCL. Apart from tumor burden, our findings indicated that the pre-treatment DLBCLRP expression was also associated with response assessment and IPI in patients with DLBCL. In view of the competition for capturing glucose/FDG between the tumor and the brain tissue, we speculated that the tumor burden of lymphoma might indirectly affect the expression of DLBCLRP through undermining the brain glucose supply. Moreover, several PET-based studies recommended that the semi-quantitative parameters of tumor metabolic burden should be independent prognostic factors for the DLBCL [38–40]. Thus, it was reasonable to evaluate the prognostic value of the brain glucose metabolic level and the expression of DLBCLRP in patients prior to any treatment. The SUV ratio of the cerebellum over the liver extracted from baseline PET data might be a predictor of progression-free survival [23]. Besides, it was suggested that low metabolic volume products in not only cerebellum but also basal ganglia were associated with a significantly poor prognosis [19]. Another study using voxel-wised analysis, however, indicated that the higher pre-treatment metabolic level in basal ganglia and thalamus might predict a relatively worse outcome in patients with extranodal natural killer/T-cell lymphoma [22]. These discrepancies can be attributed to the differences in pathological types or data processing methods between these studies. The herein proposed expression of DLBCLRP in the form of Z-transformation provided a novel covariance parameter reflecting the whole-brain metabolic pattern, and more investigations are needed to define its prognostic value and psychological symptoms implication.

Limited evidence showed that the chemotherapy-induced aberrant metabolic network mainly involved the prefrontal cortex and cerebellar areas [58]. It is still up for debate whether or not cerebral metabolic abnormalities related to the disease status of lymphoma will change after receiving chemotherapy [19, 21]. In the current study, we found that there was a significant reduction in the DLBCLRP expressions of post-treatment, compared with those of baseline. More importantly, the post-treatment declines of DLBCLRP expression were significantly positively correlated with Ann Arbor staging and IPI as well. As this is an emerging field of research, further implications of these altered brain network should be explored in patients with extracranial DLBCL or other cancer.

Several limitations of the current study should be acknowledged. Due to the retrospective study design, the assessment of cognitive function or neuropsychiatric inventory was absent. Accompanied by the more and more attention to brain function in patients with lymphoma or other cancer, the data for neuropsychiatric symptoms would be routinely collected and applied to explore its correlation with the brain functional/metabolic network. By the same token, the data of progression-free survival and overall survival are not yet available owing to insufficient follow-up. Further long-term follow-up would be essential for the reliable assertion regarding the prognostic value of DLBCLRP. In addition, though our findings revealed that the metabolic brain pattern of HL patients was distinct from that of DLBCL patients, other types of FDG-avid lymphoma (e.g., follicular lymphoma) were not investigated. It is also fascinating to determine whether there is a difference in metabolic brain pattern between lymphoma and other common solid tumors.

## Conclusion

The established DLBCLRP was characterized by the increased metabolic activity in brain regions rarely involving cerebrum and different from that of HL. The association between the expression of DLBCLRP and tumor burden might imply a potential biomarker for DLBCL staging and prognosis.

## Data Availability

The data generated during and/or analyzed in this study are not publicly available due to restrictions imposed by the administering institution, but are available from the corresponding author upon reasonable request and writing a data sharing agreement.

## Abbreviations

CMR: Complete metabolic response
DLBCL: Diffuse large B-cell lymphoma
DLBCLRP: DLBCL-related pattern
ECOG: Eastern Cooperative Oncology Group
^18^F-FDG PET/CT: ^18^F-fluorodeoxyglucose positron emission tomography-computed tomography
5-PS: 5-Point scale
HL: Hodgkin’s lymphoma
IPI: International prognostic index
MTV: Metabolic tumor volume
NHL: Non-Hodgkin’s lymphoma
NMR: No metabolic response
non-CNS: Non-central nervous system
PMD: Progressive metabolic disease
PMR: Partial metabolic response
ScAnVP: Scan Analysis and Visualization Processor
SPM: Statistical parametric mapping
SSM-PCA: Scaled subprofile model of principal component analysis
SUV: Standardized uptake value
3D: Three-dimensional
TBG: Total brain glycolysis
TLG: Total lesion glycolysis
TMTV: Total metabolic tumor volume
VOI: Volume of interest
